# Effects of cardiac rehabilitation in patients with ventricular assist devices: a scoping review

**DOI:** 10.1051/ject/2024017

**Published:** 2024-09-20

**Authors:** Nelson Esteban Portuguez Jaramillo, Angely Paola Ceron, Jose Luis Piñeros Álvarez, Eleonora Giron Ruiz, Carolina Castro Gómez

**Affiliations:** 1 Faculty of Health, Universidad Santiago de Cali Cl. 5 #No. 62-00 760035 Cali Colombia; 2 Physical Medicine and Rehabilitation E.S.E., Hospital Universitario Evaristo Garcia ESE Cl. 5 #36-00 760042 Cali Colombia; 3 Basic Sciences Department, Institución Universitaria Colegios de Colombia Unicoc-Cali 760045 Cali Colombia; 4 Coordination of Research, Innovation and Teaching Service, Clínica Colombia Cra. 46 #9c-58 760036 Cali Colombia; 5 Fundación Valle del Lili, Medicina física y rehabilitación Carrera. 98 #18-49 760032 Cali Colombia

**Keywords:** Cardiac rehabilitation, Ventricular assist devices, Oxygen consumption, Exercise tolerance, Exercise prescription, High-intensity interval training, Exercise programmes

## Abstract

*Introduction*: Ventricular assist devices represent a treatment option for patients with advanced heart failure, offering control over various haemodynamic variables. Similarly, the prescription of exercise within a cardiac rehabilitation programme for heart failure patients is recommended to reduce symptoms, and hospitalisations, improve cardiorespiratory fitness, and increase exercise tolerance. Therefore, exercise prescription can impact those with ventricular assist devices. Given the limited evidence on exercise-based cardiac rehabilitation programmes for this population, this review aims to describe the most commonly used strategies and their health benefits when physical exercise is included in a cardiac rehabilitation programme for patients with ventricular assist devices. *Materials and methods*: An exploratory review was conducted through searches in the databases: PubMed, SCOPUS, PeDro, and ScienceDirect. The search was limited to studies published between 2013 and 2023. Filters were applied independently by title, abstract, and full text. The included articles were analysed based on the description of the types of cardiac rehabilitation strategies used in patients with ventricular assist devices. *Results*: Seven articles were included. Each programme employed a cardiopulmonary exercise test before prescribing physical exercise. The most commonly used strategy was aerobic exercise, predominantly high-intensity interval training (HIIT) with intensities close to 90% of peak VO_2_, followed by continuous moderate-intensity exercise. Limb strength exercises were included in three programmes. *Conclusions*: The analysed literature suggests that cardiac rehabilitation in patients with ventricular assist devices is safe and can provide benefits in cardiorespiratory fitness and exercise tolerance. High-intensity interval training is identified as an appropriate strategy for achieving results, offering short-term improvements.

## Introduction

In healthy individuals, the increased peripheral demand for oxygen during physical activity is met with an increase in cardiac output, facilitated by physiological variables such as preload, ventricular contractility (Frank-Starling mechanism), heart rate, and afterload [1]. However, in patients with advanced or end-stage heart failure (HF), there is a significant compromise in meeting cardiometabolic demand, resulting in reduced cardiac output, hypoperfusion, increased intracardiac pressures, and severe deterioration of functional capacity [2, 3].

With a prevalence of HF of at least 2% in developed countries, the condition has a significant public health impact, affecting synthetic health indicators and presenting a long road to heart transplantation as the reference intervention. Advances in ventricular assist devices (VADs) have allowed them to be considered as an alternative for candidates awaiting transplantation, during the transplant process, and for recovery [3]. The goal of VADs is to restore tissue perfusion and enhance systemic blood supply. Different types of VADs exist with varying mechanisms of action, classified according to the type of support provided, either left ventricular, right ventricular, or biventricular. Additionally, mechanical circulatory systems are classified by usage duration: short-term devices include intra-aortic balloon pumps, IMPELLA, TANDEM-Heart, and CentriMag, while long-term devices include HeartMate II and, more recently, HeartMate III [3].

Scientific evidence strongly recommends cardiac rehabilitation (CR) for patients diagnosed with HF to improve functional capacity, quality of life, and reduce mortality risk [4]. The exercise prescription (EP) component within CR has demonstrated physiological changes that contribute to the favourable outcomes of CR [5].

However, EP can pose a significant challenge in HF patients with VADs due to the unique pathophysiological characteristics of this population. For instance, in patients with VADs, the ability of each device to adjust the flow rate according to workload during exercise remains enigmatic and depends on its flow control mechanism. Therefore, there is currently no clear consensus on intervention strategies for EP in patients with VADs [6, 7].

Consequently, intervention strategies aimed at improving physiological parameters related to adequate cardiovascular function and meeting metabolic demands in patients with VADs should be based on quantifiable and measurable objectives.

This scoping review aimed to explore scientific reports on the EP used in adult patients with VADs participating in a CR programme, focusing on exercise modalities and observable changes in physiological variables related to symptom reduction, aerobic capacity improvement, and cardiorespiratory fitness.

## Materials and methods

A scoping review was conducted following the methodology described in the Joanna Briggs Institute Manual [8], the protocol presented by Arksey and O’Malley [9], and the improvements suggested by Levac et al. [10]. This review included defining the research question, conducting systematic searches, study selection, review, and qualitative synthesis.

The review addressed the question: What are the EP strategies used in CR for patients with ventricular assist devices (VADs)? The inclusion criteria were as follows: population: Patients over 18 years old with recently implanted VADs. Concept: Types of EP strategies implemented in CR. Search Limits: Epidemiological designs including controlled and uncontrolled clinical trials, prospective cohorts, including single-blind, double-blind, and/or randomised studies, published between 2013 and 2023 in English.

During the systematic search, keywords such as “ventricular assist device,” “cardiac rehabilitation heart transplant,” and “exercise training” were included, along with the following search equation: (ventricular assist device OR Centrimag OR VAD OR HeartMate II) AND (cardiac rehabilitation OR exercise OR exercise training) AND (heart transplant OR left ventricular failure OR right ventricular failure OR biventricular assist device OR heart pump OR implantable ventricular assist system).

Two researchers independently conducted systematic searches in the databases: PubMed, SCOPUS, ScienceDirect, and PEDro. Notably, PEDro was highlighted for its value as a comprehensive and reliable source providing high-quality evidence in the field of rehabilitation, including CR.

**Figure 1:**
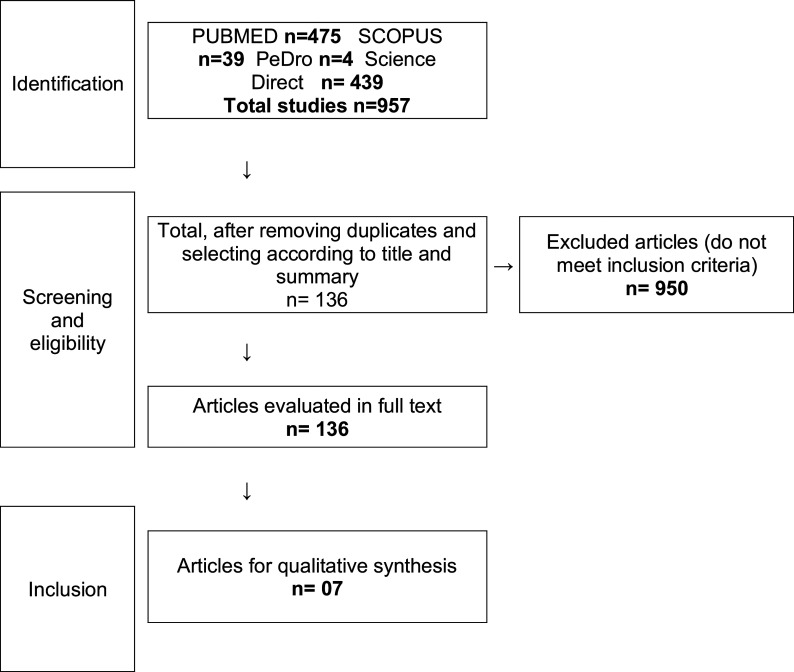
Study selection flowchart.

After removing duplicates, two researchers independently reviewed the titles and abstracts resulting from the search and included studies that described the types of training during CR in the previously described population. Subsequently, the full text of 136 studies was reviewed to determine how each responded to the research question. Following this, a consensus among all researchers led to the inclusion of seven articles. Data extraction was then performed, capturing study aspects in a digital spreadsheet (authors, year of publication, number of patients, type of training, description of training, intervention duration, and post-intervention changes) (Figure 1).

In the final review stage, the content of the included studies was synthesised into Table 1, and the analysis focused on the different strategies used and post-intervention changes in CR programmes. This final point was of high importance for the authors, who sought to provide a theoretical basis for the variation in physiological parameters through EP.

**Table 1:** Characteristics of the included studies.

Authors	Moreno et al.		Kerrigan et al.	Scaglione et al.		Alvarez Villela et al.	Schmidt et al.	Marko et al			Schmidt et al
Year of publication	2020		2014	2021		2020	2018	2021			2017
Number of patients	Total participants *n* = 22		Total participants *n* = 26	Total participants n = 50		Total participants *n* = 12	Total participants *n* = 10	“Total participants *n* = 41 patients with LVAD”			Total participants *n* = 68 patients with LVAD
			Distributed in: Control group *n* = 8 (1 does not complete the program); CR Group *n* = 18 of which 16 complete training.								
Intervention duration	13 weeks, 3 times per week		6 weeks, Frequency: 3 times a week. In both groups, follow-up calls were made in week 2, 4, and 6 (new signs or symptoms, medications, hospitalizations).	Hospitalization period (T0): from 15 to 62 days, approximately 4 weeks in-hospital.	Discharge Time (T1) 2 sessions a day for 6 days a week.	15 sessions 5 weeks	Duration: 3 weeks, 22 days and each session corresponded to 30 min.	Duration: 32 ± 6 days of rehabilitation			Duration: 3–5 weeks
							Follow-up of 482 days after device implantation.				
Training description	HIIT Group	MICT Group	CR Group	LVAD Group *n* = 25	HTx Group *n* = 25	HIIT Group	Interval training: Bicycle, at the beginning of the CR average of 10/25 W at the beginning of the CR and at the end to an average of 14/36 W.	MMII Strength Training	Aerobic training	Treadmill	MMII Muscle Endurance Training
Training description	They carried out an evaluation at the beginning and at the end of the program with a 6-minute walk test. Training 4 sets of 4 min with intensity of 80%–90% VO_2_ alternating with 3 min lower intensity 50%–60% VO_2_.	Carry out evaluation at the beginning and end of the program with a 6-minute walk test. Training for 28 continuous minutes with a reserve of 50%–60% VO_2_.	Physical training of 18 sessions of aerobic exercise between 60% and 80% of the HR reserve. It included walking on a treadmill, stationary bicycle, arm ergometer, recumbent stepper) 30 min at 60% of HR max 80%.	Initial evaluation 6MWT, Heart Rate and Borg.	HTx group: They underwent the same CR program as LVAD patients	Each training session lasted 30 min: 3 min warm-up and six 30-s high-intensity intervals, each followed by a 4-min active recovery period.	Bicycle, at the beginning of the RC average 10/25 W at the beginning of the RC and at the end up to an average of 14/36 W.	MMII strength training: Leg press, leg extensor, leg flexor, lower limb abductor, lower limb adductor, 2 sets of 12 repetitions each.	Aerobic training: Bicycle ergometer, 3-minute intervals of cycling without load at the beginning and end of the session to warm up and cool down.	Hikes: Trails that covered different distances and elevations in different periods of time.	Frequency: 5 and 7 days per week.
				Interval training (Bicycle/treadmill) and progression to continuous exercise 40 min per session.		For the first three sessions (“induction phase”), the prescribed workloads were 40% PPO warm-up, 80% PPO intervals, and 30% PPO cool-down periods.					Resistance exercise was especially focused on muscular endurance.
											Lower extremity training (3 sets of 20 repetitions) using medical exercise machines (e.g., leg curl, leg extension, and leg press) or small exercise tools (e.g., theraband and dumbbells).
				Strength training: 1 set of 12 repetitions of 5 muscle groups MMSS and MMII. Aerobic exercises: walking and/or cycling 60–70% of the maximum oxygen consumption measured in the stress test. Respiratory exercises: Respiratory incentive.		Workloads were increased in the fourth training session to 50% PPO warm-up, 100% PPO high-intensity intervals, and 40% PPO recovery periods.		Gymnastic training: coordination, strength and balance training.			Ergotherapy (if necessary) and exercise therapy (including resistance training on a bicycle ergometer) were generally performed 3–5 days per week.
											The monitored bicycle training in most cases was performed using the interval method with 20 s of high intensity tracking followed by 40 s of low intensity.
Post-intervention changes	Carry out evaluation at the beginning and end of the program with a 6-minute walk test. Training for 28 continuous minutes with a reserve of 50%–60% VO_2_	Improvement in treadmill stress test from 7.9 to 11.0 min.	“There were no significant differences in T0 and T1 at 6MWT in patients with VADI and HTX			Improvement in V̇O2 at the ventilatory threshold of 7.1 to 8.5 ml/kg/min.	Visit 1: 6MWT walking distance > 367 to 449 meters. VO_2_ max 10.0 to 11.9 ml/kg/min. Maximum load increased from 62.4 to 83.0 W. Handgrip strength test from 29.2 to 34.7 kg without statistically significant changes	Muscular strength in all trained muscle groups 26.6 ± 11.9 kg 33.6 ± 15.2 in leg press	Improvement in the intensity of the bicycle ergometer: 2.0 ± 1.9 vs. 6.2 ± 2.8	VO_2_ increase 11.3 ± 4.1 ml/min/kg vs. 14.5 ± 5.2	“6-minute walk distance was significantly improved during CR (325 ± 106 to 405 ± 77 m; *P* < 0.01).
		Oxygen consumption of 13.6 to 15.3 ml/kg/min.	Changes were evident in: HB: 10.2–10.8 Average corpuscular volume: 89–89.8 Creatinine: 0.85–0.99 Red blood cells: 3.59–3.66			LV end-diastolic volume 159–168 ml					The average maximum workload achieved was 62.2 ± 19.3 W (38% of predicted values).
		Improvement in 6MWT 350.1 ± 64.7 to 402.4 ± 89.3									Mean cardiopulmonary exercise capacity (relative maximal oxygen consumption) was 10.6 ± 5.3 ml/kg/min (37% of predicted values).

Abbreviations: *n*: Number of participants, HIT Group: High Intensity Interval Training, MICT: Moderate Intensity Continuous Training, VO_2_ Peak: Maximum Oxygen Consumption, CR: Cardiac Rehabilitation, HR: Heart Rate, 6MWT: 6 Minute Walk, T0: Functional and psychological tests at admission, T1: Functional and psychological tests at discharge, LVAD Group: Left Ventricular Assist Device, HTX Group: Heart Transplant Patients, LVAD: Left Ventricular Assist Device, VADI: Assist Device left ventricular, HB: Hemoglobin, PPO: Maximum power output, VI: Left ventricle, W: Watts, Mts: Meters, MMII: Lower limbs.

## Results

### Characteristics of the studies and target population

The included studies were conducted in North America, Europe, and Oceania, with the oldest study published in 2014 [11]. The studies by Kerrigan et al. [11], Moreno et al. [12], and Scaglione et al. [13] were designed as experimental studies, whereas the studies by Alvarez Villela et al. [14], Schmidt et al., and Marko et al*.* [15–17] opted for a quasi-experimental design. A total of 226 participants were analysed, including 149 males and 32 females, excluding the studies by Kerrigan and Moreno where the population specifics were not provided.

### Strategies used during cardiac rehabilitation and post-intervention changes

Three studies employed aerobic resistance training, specifically high-intensity interval training (HIIT). Moreno et al. [12] prescribed sessions of four sets, each lasting 4 min, with an intensity of 80–90% of VO_2_ peak, alternating with 3 minat lower intensities around 50% of VO_2_ peak. This resulted in a significant improvement in VO_2_ peak from 15.6 to 18.4 ml/kg/min compared to the control group, which performed continuous training for 28 min at 50–60% of VO_2_ peak, increasing from 16.2 to 17.2 ml/kg/min. Alvarez Villela et al. [14] established a protocol with progressive intensity increments, starting at 80% workload with 30% recovery periods, and increasing to 100% with 40% recovery by the fourth session, showing a significant improvement in VO_2_ peak from 7.1 to 8.5 ml/kg/min and left ventricular end-diastolic volume from 159 to 168 ml.

Similarly, Schmidt et al. [15] implemented a HIIT protocol with intensity measured in watts, starting at 10/25 W and ending at 14/35 W. Significant improvements were reported in the six-minute walk test (6MWT) distance from 367 to 449 m, VO_2_ peak from 10.0 to 11.9 ml/kg/min, maximum workload from 62.4 to 83.0 W, and handgrip strength from 29.2 to 34.7 kg, though these were not statistically significant.

Two additional studies also used aerobic resistance training. Kerrigan et al. [11] established a six-week protocol with three sessions per week, involving 18 sessions of aerobic exercise at 60-80% of maximum heart rate. Improvements were seen in treadmill test duration from 7.9 to 11.9 min, oxygen consumption from 13.6 to 15.3 ml/kg/min, and 6MWT distance from 350.1 ± 64.7 to 402.4 ± 89.3 m compared to no EP or physiological improvements in the control group. Marko et al. [16] implemented a 32 ± 6-day strength training protocol, focusing on lower limbs, dividing participants based on underlying heart disease, age, and post-operative conditions. In one group (*n* = 15), ergo-spirometry was performed at the start and end of CR, showing an increase in VO_2_ peak from 11.3 ± 4.1 to 14.5 ± 5.2 ml/min/kg. Thirty-nine patients performed lower limb strength training, with an average of 6.4 sessions, showing a significant increase in the weight lifted across all evaluated muscle groups. Other improvements included exercise duration from 14 ± 2 min to 19 ± 4 min and ergometer bike intensity from 2.0 ± 1.9 W to 6.2 ± 2.8 W.

On the other hand, Schmidt et al. [17] used a muscular endurance rehabilitation protocol lasting 3–5 weeks, 5–7 days per week, with three sets of 20 repetitions, including lower limb training and ergometer biking. Significant improvements were noted in the 6MWT distance from 325 ± 106 m to 405 ± 77 m. Additionally, they evaluated the peak workload in a cardiopulmonary exercise test (CPET) at the end of CR, reporting an average peak workload of 62.2 ± 19.3 W, corresponding to 38% of the total calculated for the population, and an average relative VO_2_ peak of 10.6 ± 2.9 ml/kg/min, corresponding to 37% of the predicted VO_2_ peak.

The average intervention duration was 5.57 weeks for the seven included studies, totalling an average of 25.42 sessions. The longest protocol was by Moreno et al. [12] with 12 weeks and 36 sessions, whereas Alvarez Villela et al. [14] conducted 15 sessions over 5 weeks, the shortest protocol. One study conducted its intervention in an inpatient setting with two sessions per day over 4 weeks, totalling 48 sessions [13]. Three of the seven studies had participants engage in sessions three times a week [11, 12, 14], while the others had near-daily sessions.

Notably, Scaglione et al. [13] included laboratory tests such as haemoglobin levels, mean corpuscular volume, creatinine, and other serological follow-ups pre-intervention and post-discharge, confirming the infrequency of events such as bleeding and no significant serological findings potentially influenced by CR. Schmidt et al. [17] supplemented their protocol with measures of anaerobic thresholds, 12-lead ECG tracings, and lactate levels, evaluating cardiac electrical activity and lactate exportation in response to increased metabolic demands.

## Discussion

This study reviewed 07 studies employing various CR strategies in patients who underwent VAD implantation. These devices serve as a treatment option for patients unresponsive to traditional heart failure management and those awaiting heart transplants. To date, over 22,000 VADs have been implanted in North America, with more than 2,500 of these devices being implanted annually [18]. Cardiopathies in general represent a significant percentage of mortality and morbidity in the population, and they are among the primary contributors to disability and reduced quality of life [19, 20]. A VAD patient can maintain haemodynamic control and adequate perfusion; however, the functional deterioration caused by their underlying condition results in severe symptoms during exertion, hindering the completion of certain tasks.

In this regard, CR is a multidisciplinary set of interventions, one of which is physical exercise [21]. The improvement in functional capacity provided by CR reduces symptoms, hospitalisations, and complications associated with heart disease [21, 22]. Designing a CR programme with the correct EP is indicated for VAD patients [4].

The CR programmes reviewed in these seven studies implemented cardiopulmonary exercise testing (CPET) or field tests like the 6-minute walk test (6MWT) to observe the physiological response to exercise before and after CR in VAD patients. These tests are considered the gold standard for EP [4] as they are objective and traceable over time. Moreover, their results reflect cardiorespiratory fitness, understood as the integrated capacity to transport oxygen, under adequate cardiopulmonary function, and the ability of muscle cells to utilise this oxygen [23].

Exercise intensity or workload prescriptions are derived from peak indices of various physiological variables, with the most commonly used being the percentage of maximum workload (Wmax%), as used by Schmidt et al. [15], the percentage of maximum heart rate (HRmax%), the percentage of peak oxygen consumption (VO_2_ peak%), the percentage of heart rate reserve (HRR%), and maximum oxygen consumption (VO_2_ max) [23], chosen by other authors. Given space, resource, and equipment constraints, HRmax% and HRR% are often the preferred reference values for prescribing intensities in outpatient CR programmes [23]. It is also advisable to continuously monitor physical effort and perceived dyspnoea, as these reflect the patient’s response to the intervention and serve as subjective indicators of progression in exercise intensity [24]. These measurements have proven to be applicable to VAD populations [23].

Exercise parameters such as intensity, frequency, duration, and volume physiologically impact the body by inducing changes in the cardiovascular system, such as increased stroke volume, improved cardiac output, reduced peripheral vascular resistance, and maintenance of adequate blood cell populations. In the respiratory system, it improves respiratory muscle function, facilitates proper breathing patterns, and enhances lung volumes and capacities, thus benefiting oxygen transport to tissues [25]. Additionally, skeletal muscle effects of CR include greater muscle fibre recruitment, increased capillary density, enhanced mitochondrial expression, stimulation of anabolic signals, and profiles of type I and type IIa muscle fibres, with a reduction in type IIb fibres, resulting in an adequate oxygen extraction capacity [25, 26], ultimately leading to an increase in VO_2_.

Studies by Moreno et al., Kerrigan et al., Scaglione et al., Alvarez Villela et al., Schmidt et al., and Marko et al. favoured aerobic exercise [11–16], supported by clinical practice guidelines [4, 6]. Traditionally, this training has been conducted under a moderate-intensity continuous training (MICT) modality [27, 28], with two studies opting for this modality: Kerrigan et al. [11] and Marko et al. [16]. In contrast, four studies, Moreno et al. [12], Scaglione et al. [13], Alvarez Villela et al. [14], and Schmidt et al. [15], preferred the high-intensity interval training (HIIT) modality, a more contemporary strategy.

On the other hand, MICT is characterised by maintaining the same exercise intensity for a prolonged period. The European Association of Preventive Cardiology defines this intensity as a range between 40% and 69% of VO_2_ max, 55% and 74% of HRmax, or 40% and 69% of HRR [29]. Meanwhile, HIIT involves training at high intensities for short periods, ≥ 90% of VO_2_ max, with longer recovery periods at lower intensities [23].

There is limited scientific evidence regarding the most recommended modality for CR services in VAD patients. In heart failure and coronary artery disease patients, HIIT has demonstrated physiological benefits related to VO_2_ max and VO_2_ peak [30], which are indicators of improved cardiorespiratory fitness and an adequate response to cardiometabolic demands [23]. CR programmes implementing HIIT have shown greater increases in VO_2_ peak% at 4 weeks compared to MICT; however, when following up on this variable after a year, HIIT is slightly superior to MICT to the point where they can yield similar VO_2_ peak% results [31]. This situation may coincide with the results of Moreno et al. [12], whose study lasted 12 weeks with a frequency of 3 sessions per week. Other studies with similar frequency, duration, and HIIT modality are those proposed by Kerrigan et al. [11], Alvarez Villela et al. [14], and Schmidt et al. [15], demonstrating changes in physiological variables, although not compared with MICT populations. Marko et al*.* [16] used both modalities, with a greater emphasis on MICT.

Interventions in VAD patients can be performed in both inpatient and outpatient settings, should be individualised, prescribed with a prior CPET, continuously monitored, and followed-up on patient perception [4]. As noted by Alvarez Villela et al. [14]*,* in studies with small populations, HIIT application in VAD patients has shown positive results in improving physiological variables within a few weeks, with a frequency of 3 sessions per week [14]. Significant differences have been identified in favour of HIIT for VO_2_ peak compared to MICT, but no differences have been found in 6MWT and patient perception scales [14]. Further research is necessary to establish precise indications in CR protocol designs.

Incorporating muscle strengthening exercises is considered in CR designs for VAD patients [4], with the 1RM test recommended beforehand to prescribe workloads, and the training should involve approximately 11–14 repetitions. Marko et al*.* [16] and Schmidt et al. [17] demonstrated that muscle strength training can be applied to VAD patients, increasing skeletal muscle strength. Overall, the benefits of CR can manifest in patients as adaptations to moderate to intense physical activities, providing sufficient quality to perform various daily tasks, with a perceived reduction in physical effort and dyspnoea, representing an optimal complement for VAD patients.

Only the study by Marko et al. [16] reported a single complication associated with EP, characterised by sustained ventricular tachycardia in one patient during ergometric cycling. The authors emphasise the importance of adapting training programmes to the patient’s capabilities and clinical conditions to ensure a safe environment.

In this context, the Exercise Physiology and Training Committee and the Advanced Heart Failure Committee of the Heart Failure Association of the European Society of Cardiology presented guidelines and safety measures to reduce the risk of adverse events during EP for VAD patients [5]. They emphasise the appropriate evaluation of symptoms, clinical signs, and functional capacity to identify the most appropriate intervention, along with the proper selection of workloads and individualisation of the patient for CR. Staying below the predetermined ventilatory anaerobic threshold [5] is crucial. Continuous monitoring during each session, patient supervision, clinical adaptation, and proper VAD functioning are also important.

### Limitations

It is noteworthy that the studies conducted have designs that include groups of modest sizes, possibly due to the difficulty in accessing this population, necessitating further research of this nature to provide more statistical certainty regarding intervention modalities and their benefits. The indication of CR in patients with VAD is increasing in several countries; however, this study was limited to research written in English, potentially excluding high-quality research published in other languages.

Some reports lack clarity in EP parameters, such as volume, load, intensity, and frequency, creating a gap in the specificity of prescription for participant interventions.

### Conclusions

All studies employed CPET or similar tests before and after the implementation of a CR program in VAD patients to screen participants and be objective with EP parameters, as well as to observe changes before and after CR. VO_2_peak is perhaps the most evaluated physiological parameter, reflecting cardiorespiratory fitness. Exercise intensities can be calculated with reference to VO_2_peak, VO_2_ max, Wmax, HRmax, and HRR. In six studies, an aerobic training strategy was chosen, while one opted for a muscle-strengthening modality. HIIT was the most used training modality, increasing VO_2_ peak in a short period compared to MICT. CR can be implemented in-hospital or on an outpatient basis, proving to be safe, with a low complication rate. More studies are needed to strengthen the field of CR in VAD patients.

## Data Availability

All research data related to this study are included in the study.
